# Use and experience with six-monthly paliperidone in the Campo de Gibraltar area. Descriptive study.

**DOI:** 10.1192/j.eurpsy.2023.2135

**Published:** 2023-07-19

**Authors:** C. M. Gil Sánchez, J. A. Salomón Martínez, E. Corbacho Navarro

**Affiliations:** 1 Psychiatry, Sistema Andaluz de Salud (SAS), ALGECIRAS; 2 Psychiatry, Sistema Andaluz de Salud (SAS), Cádiz, Spain

## Abstract

**Introduction:**

Long-acting injectable antipsychotics have demonstrated advantages over therapeutic adherence and can reduce the rates of relapses and due to treatment discontinuation. The novel presentation of paliperidone palmitate six-month (PP6M) can simplify the treatment to two injections per year.

**Objectives:**

The purpose of the present research is to describe the profile of patients receiving this novel treatment in our area. For this, a descriptive study has been carried out.

**Methods:**

We have collected and analyzed data from a total of 8 patients from the global long-acting injectable nursing registry in our area. The data collection was from May 2022 to October 2022.

**Results:**

**Table:** 

***ID Patient***	**Age**	** Gender**	**Medical comorbidities**	** Social support**	**Adherence to previous LAI**
*EP001AGC*	52	M	No	-	Yes
*EP002EGA*	53	M	No	Low	No
*EP003ESL*	45	F	Yes HIV, HCV, dyslipidemia,	Enough	Yes
*EP004ACG*	60	M	Yes Hypertension, dyslipidemia	Good	Yes
*EP005DCP*	52	M	Yes COPD	Enough	Yes
*EP006ATT*	47	M	No	Enough / Low	Yes
*EP007AH*	40	F	Yes Tension headache	Enough	Yes
*EP008IAR*	66	F	Yes Type 2 diabetes mellitus, hypertension, hyperuricemia	Enough	Yes

**Table:** 

ID Patient	Diagnosis	Refractory positive symptoms	Last H.	Polypharmacy	Previous injection	Injection date / Dose	H. / Side Effects
EP001AGC	Paranoid schizophrenia	-	08/03/2014	No	PP3M 525mg	17/05/22 1.000 mg	No
EP002EGA	Schizoaffective disorder	Yes	19/08/2022	Yes Valproic acid 1.000mg	PP1M 150mg (once)	13/09/22 1.000mg	No
EP003ESL	Paranoid schizophrenia	No	17/04/2019	Yes Olanzapine 10mg BZD	PP3M 525mg	10/08/22 1.000mg	Sedation (low)
EP004ACG	Paranoid schizophrenia	No	-	Yes Quetiapine 50mg	PP3M 525mg	16/09/22 1.000mg	No
EP005DCP	Paranoid schizophrenia	No	16/01/2004	Yes Olanzapine 20mg BZD	PP3M 525mg	11/10/22 1.000mg	No
EP006ATT	Persistent delusional disorder	Yes	-	No	PP3M 525mg	19/09/22 1.000mg	No
EP007AH	Paranoid schizophrenia	No	2017	No	PP3M 525mg	03/08/22 1.000mg	No
EP008IAR	Persistent delusional disorder	Yes	-	Yes BZD	Paliperidone oral 9mg and later PP3M 350mg (twice)	18/10/22 1.000mg	No

Fig. 1: Sociodemographic characteristics and Fig. 2: Clinical characteristics.

**Conclusions:**

None of the patients required hospitalization at the time of the study, although this work team considers that it is early to make conclusions in this regard. No serious or minor adverse effects were reported in any of the cases during the time of the investigation, apart from one case of mild sedation.

The clinical characteristics of most patients were psychopathological stability and good adherence to previous treatment. Although this study shows that the drug was also used in patients who did not meet these characteristics, specially one case of poor social support. The data collected show that the profile of the patient in whom the drug has been prescribed can be varied and broad.

**Disclosure of Interest:**

None Declared

